# CT-Derived Aortic Plaque Characteristics Predict MRI-Detected Silent Cerebral Infarction after Total Aortic Arch Replacement

**DOI:** 10.5761/atcs.oa.25-00215

**Published:** 2026-04-14

**Authors:** Fumio Yamana, Kazuo Shimamura, Takayuki Shijo, Koichi Maeda, Kizuku Yamashita, Ryoto Sakaniwa, Shigeru Miyagawa

**Affiliations:** 1 Department of Cardiovascular Surgery, The University of Osaka Graduate School of Medicine, Suita, Osaka, Japan; 2 Department of Cardiovascular Surgical Technology Innovation (CaSTi), Osaka University Graduate School of Medicine, Suita, Osaka, Japan; 3 Department of Social and Environmental Medicine, The University of Osaka Graduate School of Medicine, Suita, Osaka, Japan

**Keywords:** low-attenuation plaque, total arch replacement, cerebral infarction, diffusion-weighted MRI, silent ischemia

## Abstract

**Purpose:**

Silent cerebral infarctions are common after aortic arch surgery; however, the predictive value of preoperative computed tomography (CT)-derived plaque characteristics remains unclear. We investigated the incidence, distribution, and risk factors for new cerebral infarction lesions (NCILs) after total aortic arch replacement (TAR), focusing on low-attenuation plaque (LAP, 0–60 Hounsfield units [HU], a surrogate of lipid-rich vulnerable plaque) burden.

**Methods:**

Among 82 consecutive TAR patients, 41 underwent both pre- and postoperative brain diffusion-weighted magnetic resonance imaging (MRI). Clinical profiles, CT-derived atheroma grade and plaque attenuation, operative details, and outcomes were compared between NCIL-positive and NCIL-negative groups. The primary multivariable model simultaneously included arch atheroma grade and LAP area, adjusted for age and sex.

**Results:**

NCILs were detected in 25/41 patients (61%): 23 silent and 2 symptomatic. All NCILs exhibited embolic imaging features without watershed or hypoperfusion patterns. NCIL-positive patients had significantly greater arch LAP area (63.9 vs. 17.7 mm^2^, *p* <0.01). On multivariable analysis, arch LAP remained the only independent predictor (OR per 10 mm^2^, 3.01; 95% confidence interval [CI] 1.50–8.75; *p* = 0.012), whereas atheroma grade was not.

**Conclusion:**

More than half of TAR patients developed MRI-detected, predominantly silent NCILs. Preoperative arch LAP was the sole independent predictor. LAP assessment may refine intraoperative risk stratification and guide tailored neuroprotective strategies.

## Introduction

Aortic arch surgeries are associated with the highest risk of intraoperative stroke, with rates ranging from 2% to 16%.^1,2)^ Intraoperative cerebral infarction affects long-term postoperative survival, and outcomes vary according to embolic territory.^3)^ Diffusion-weighted magnetic resonance imaging (DW-MRI) detects acute cerebral infarction, including silent lesions, which have been linked to neurological dysfunction (e.g., cognitive and neuromotor function) late after onset,^4)^ and whose severity is influenced by lesion location and number.^4)^

In the contemporary era of selective cerebral perfusion, improvements in cerebral perfusion strategies have substantially reduced hypoperfusion-related brain injury.^5,6)^ Nevertheless, clinically relevant postoperative cerebral infarction after total aortic arch replacement is generally thought to involve embolic mechanisms, potentially influenced by aortic manipulation and cannulation strategies.^7)^

However, the MRI-defined incidence and distribution of silent lesions after total aortic arch replacement (TAR) remain poorly characterized. Recent studies have focused on the qualitative characteristics of aortic plaques, particularly the presence of low-attenuation plaques (LAP), as assessed by computed tomography (CT).^8,9)^ LAP, defined by low Hounsfield units (HU), is considered a surrogate marker of vulnerable plaques and is associated with embolic risk in coronary and carotid diseases.^10,11)^

This study aimed to evaluate the frequency and distribution of DW-MRI-detected cerebral infarctions after TAR and analyzed risk factors with a focus on LAP burden.

## Materials and Methods

### Ethical statement

This study was conducted in accordance with the principles of the Declaration of Helsinki. The Osaka University Hospital Clinical Research Ethics Committee approved this study and the publication of its data (approval number: 16105-5; approval date: October 4, 2024). The requirement for written informed consent from each patient was waived owing to the retrospective nature of the study.

### Study population and design

**Fig. 1:**
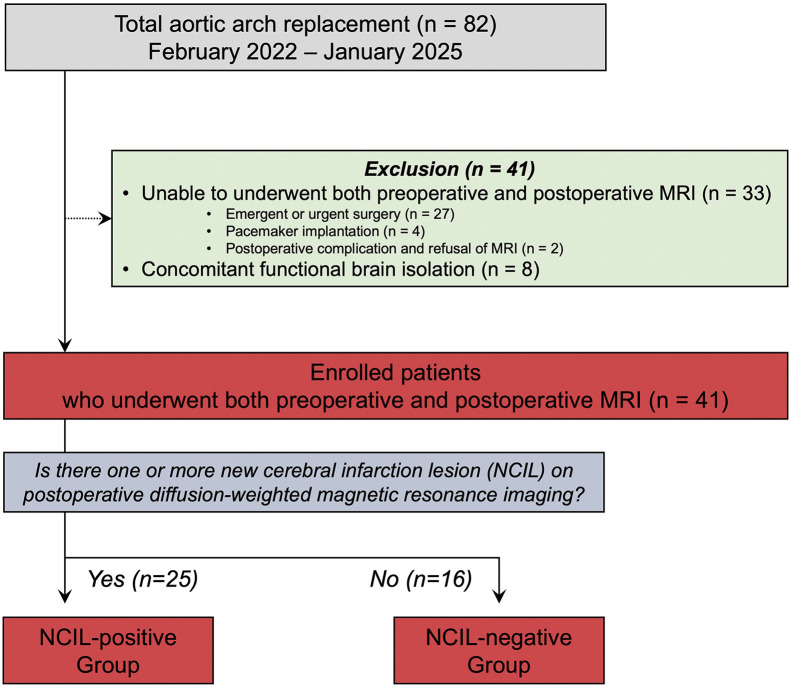
Flow diagram of patient selection. Forty-one patients undergoing TAR with both pre- and postoperative MRI were analyzed and stratified by NCIL status. TAR: total aortic arch replacement; MRI: magnetic resonance imaging; NCIL: new cerebral infarction lesion

Patient data were retrospectively collected from medical records. A total of 82 patients underwent TAR for aortic arch pathology at our institution between February 2022 and January 2025. Pre- and postoperative brain MRI data were available for 41 patients (**Fig. 1**). Patients were excluded if they could not undergo brain MRI due to emergent or urgent surgery (n = 27), the presence of an implanted mechanical device (e.g., pacemaker) (n = 4), non-neurological early death (n = 1), or refusal to undergo MRI (n = 1). In addition, patients who underwent concomitant functional brain isolation (n = 8) were excluded because flow-modifying strategies would confound the natural incidence and distribution of cerebral infarctions. Patients were classified into 2 groups based on the presence or absence of new cerebral infarction lesions (NCILs) on postoperative DW-MRI: NCIL-positive and NCIL-negative. Risk factor analysis for NCIL was also performed.

### Treatment strategy and surgical procedures

Selective cerebral perfusion is routinely performed for open TAR. Cardiopulmonary bypass was established via the right axillary and femoral arteries with systemic cooling to a bladder temperature of 25°C. Alternative inflow configurations (e.g., ascending aortic cannulation and/or femoral-only inflow) were selected based on patient-specific anatomical considerations. After clamping the brachiocephalic artery, the aorta was opened under circulatory arrest, and bilateral antegrade cerebral perfusion was achieved via 12-Fr balloon catheters inserted into the left common carotid and subclavian arteries. Standard continuous suturing with felt strip reinforcement was performed for the distal aortic anastomosis. For more distal diseases extending to the descending thoracic aorta, a relocation technique using a short elephant trunk was used, followed by completion of staged thoracic endovascular aortic repair (TEVAR).

### Brain MRI

All MRI examinations were performed on a 3.0-Tesla system (GE Healthcare, Little Chalfont, UK). Sequences included DW-MRI and T2-fluid-attenuated inversion recovery (FLAIR) (repetition time/echo time 3200/82 ms; field of view, 220 mm; matrix, 128 × 128; slice thickness, 5 mm; interslice gap, 1 mm). MRI was performed preoperatively and repeated 5–14 days postoperatively, and symptomatic patients underwent MRI at the onset of symptoms.

NCILs were defined as new diffusion-restricted lesions ≥2 mm in maximal diameter that were absent on preoperative MRI. The 2-mm cutoff was chosen to improve reproducibility and reduce false-positive calls from small punctate artifacts/partial-volume effects, consistent with prior perioperative DWI studies in cardiac surgery.^12)^

Two expert radiologists, blinded to clinical and operative information, independently assessed lesion number, laterality, and vascular territory (right/left anterior; posterior [occipital, thalamus, cerebellum, brainstem]), and specifically evaluated whether lesion patterns were compatible with watershed or border-zone infarction.

### Atheroma grading and plaque properties in CT

The severity of atherosclerosis and aortic anatomy were assessed preoperatively using contrast-enhanced 1-mm slice CT images. All images were evaluated using a 3-dimensional image analysis software (AquariusNET; TeraRecon Inc., San Mateo, CA, USA). Atheroma severity in the aorta was assessed at 4 designated sites according to the 5 atheroma grades: (i) the ascending aorta at the mid-position, (ii) the aortic arch at the proximal level of zone 2, (iii) the descending aorta at the proximal level of zone 4, and (iv) the mid-descending aorta at the proximal level of zone 5.^13,14)^ A 3-dimensional CT volume analyzer (Synapse VINCENT; Fujifilm Co., Tokyo, Japan) was used to measure the aortic plaque properties. The CT values within the plaques were assessed, and areas with CT values of 0–60 HU were classified as LAP, whereas areas with CT values of 61–130 HU were classified as intermediate-attenuation plaques (IAP) according to previous reports.^15,16)^

### End points

The primary endpoint was the occurrence of postoperative NCILs on the DW-MRI. We first described the incidence, counts, and anatomical distribution and then compared the baseline, CT plaque, operative details, and early outcomes between NCIL-positive and NCIL-negative patients. Predictors of NCIL were assessed using a logistic regression analysis.

### Statistical analysis

Intergroup comparisons were conducted using the Mann–Whitney U test for continuous variables and Fisher’s exact test for categorical variables. Variables with *p* <0.10 on univariable analysis and clinically relevant factors were entered into multivariable logistic regression models. The main model simultaneously included arch atheroma grade and LAP area, adjusted for age and sex. Sensitivity analyses were conducted by adding or substituting IAP and descending aortic atheroma variables to assess the robustness of associations. Odds ratios (ORs) with 95% confidence intervals (CIs) were calculated for each outcome. All *p*-values were 2-sided, and *p* <0.05 was considered statistically significant.

Given the limited number of NCIL events, the multivariable model was intentionally kept parsimonious, including only age and sex as core covariates in addition to plaque-related variables of interest. With an events-per-variable of approximately 6, the model may be susceptible to overfitting; therefore, the results should be interpreted as exploratory rather than confirmatory. Statistical analyses were performed using JMP version 15.0.0 software (SAS Institute Inc., Cary, NC, USA).

## Results

### Patient profiles

The patients’ characteristics are shown in **Table 1**. The median age was 77 years (interquartile range [IQR], 73–79 years). Of the 41 patients, 34 (82.9%) were men. Regarding the etiology of arch pathology, 29 patients (70.7%) had degenerative aneurysms, and 12 (30.0%) had chronic type B aortic dissection. Five patients (12.2%) had a history of stroke. Regarding the patients’ characteristics, no significant differences were found between the NCIL-positive and NCIL-negative groups.

**Table 1:** Baseline characteristics of patients undergoing total aortic arch replacement

Variable	All (n = 41)	NCIL-positive (n = 25)	NCIL-negative (n = 16)	*p* Value
Age (years), median (IQR)	77 (73–79)	77 (74–80)	77 (73–70)	0.383
Male, n (%)	34 (82.9)	21 (84.0)	13 (81.3)	1.00
BSA (m^2^), median (IQR)	1.66 (1.53–1.75)	1.66 (1.51–1.75)	1.66 (1.57–1.75)	0.862
Medical history				
Hypertension, n (%)	36 (87.8)	23 (92.0)	13 (81.3)	0.362
Hyperlipidemia, n (%)	25 (61.0)	15 (60.0)	10 (62.5)	1.00
Diabetes mellitus, n (%)	8 (19.5)	3 (12.0)	5 (31.3)	0.225
Current smoker, n (%)	28 (68.3)	16 (64.0)	12 (75.0)	0.513
COPD, n (%)	16 (39.0)	10 (40.0)	6 (37.5)	1.00
Past stroke, n (%)	5 (12.2)	4 (16.0)	1 (6.3)	0.632
Coronary artery disease, n (%)	5 (12.2)	4 (16.0)	1 (6.3)	0.632
Peripheral artery disease, n (%)	4 (9.8)	2 (8.0)	2 (12.5)	0.637
Atrial fibrillation, n (%)	4 (9.8)	4 (16.0)	0 (0.0)	0.143
Cancer, n (%)	3 (7.3)	2 (8.0)	1 (6.3)	1.00
LVEF (%), median (IQR)	64.0 (60.0–70.8)	64.0 (60.0–68.8)	64.0 (57.8–73.0)	0.59
Preoperative laboratory data				
Hemoglobin (g/dL), median ((IQR)	12.9 (12.2–14.0)	13.2 (12.5–14.0)	12.5 (11.9–14.3)	0.404
Creatinine (mg/dL), median (IQR)	0.93 (0.74–1.13)	0.93 (0.74–1.19)	0.92 (0.75–1.08)	0.593
eGFR (mL/min/1.73m^2^), median (IQR)	57.2 (47.3–73.6)	56.3 (44.6–72.8)	57.3 (51.4–74.4)	0.454
Albumin (mg/dL), median (IQR)	4.1 (3.9–4.2)	4.1 (3.9–4.3)	4.1 (3.9–4.3)	0.745
Preoperative medications, n (%)				
Anti-platelet	8 (19.5)	4 (16.0)	4 (25.0)	0.689
Anticoagulation	4 (9.8)	4 (16.0)	0 (0.0)	0.143
Statin	19 (46.3)	13 (52.0)	6 (37.5)	0.522
Aortic pathologies, n (%)				1.00
Degenerative aneurysm	29 (70.7)	18 (72.0)	11 (68.8)	
Dissection	12 (29.3)	7 (28.0)	5 (31.3)	
Aneurysm diameter (mm), median (IQR)	55.0 (53.0–62.5)	55.0 (53.0–64.0)	55.0 (52.3–59.3)	0.413
Bovine arch, n (%)	1 (2.4)	1 (4.0)	0 (0.0)	1.00

Continuous variables are presented as medians (interquartile ranges), and categorical variables as numbers (percentages).

NCIL: new cerebral infarction lesion; BSA: body surface area; COPD: chronic obstructive pulmonary disease; LVEF: left ventricular ejection fraction; IQR: interquartile range

### Aortic plaque characteristics

The plaque morphology and compositional characteristics are presented in **Table 2**. Patients who developed NCILs had significantly higher grades of atheroma in the mid-descending thoracic aorta (grades 4 and 5: 48.0% vs. 6.3%, *p* <0.01). LAP area in the ascending aorta (28.0 vs. 12.9 mm^2^, *p* = 0.032) and particularly in the aortic arch (63.9 vs. 17.7 mm^2^, *p* <0.01) was greater in NCIL-positive patients. In addition, the IAP area in the aortic arch was larger in the NCIL-positive group (63.2 vs. 50.1 mm^2^, *p* = 0.024).

**Table 2:** Preoperative aortic plaque characteristics in patients with and without NCIL

Variable	All (n = 41)	NCIL-positive (n = 25)	NCIL-negative (n = 16)	*p* Value
Atheroma grade, n (%)				
Ascending aorta				0.059
Grades 1–3	31 (75.6)	16 (64.0)	15 (93.8)	
Grades 4 and 5	10 (24.4)	9 (36.0)	1 (6.3)	
Aortic arch				0.218
Grades 1–3	23 (56.1)	12 (48.0)	11 (68.8)	
Grades 4 and 5	18 (43.9)	13 (52.0)	5 (31.3)	
Proximal descending aorta				0.120
Grades 1–3	33 (80.5)	18 (72.0)	15 (93.8)	
Grades 4 and 5	8 (19.5)	7 (28.0)	1 (6.3)	
Mid descending aorta				<0.01
Grades 1–3	28 (68.3)	13 (52.0)	15 (93.7)	
Grades 4 and 5	13 (31.7)	12 (48.0)	1 (6.3)	
LAP area (0–60 HU), mm^2^ (IQR)				
Ascending aorta	23.8 (9.5–46.6)	28.0 (16.6–59.3)	12.9 (5.1–30.3)	0.032
Aortic arch	25.0 (17.2–65.0)	63.9 (24.4–80.7)	17.7 (10.3–24.0)	<0.01
Proximal descending aorta	17.5 (7.6–59.2)	17.5 (8.6–63.7)	17.8 (1.4–29.0)	0.334
Mid descending aorta	39.1 (19.9–96.5)	51.8 (21.3–128.7)	25.2 (19.0–49.8)	0.107
IAP area (61–130 HU), mm^2^ (IQR)				1.00
Ascending aorta	60.4 (41.8–83.9)	62.6 (42.5–83.3)	58.2 (39.0–86.7)	0.889
Aortic arch	62.0 (44.5–83.2)	63.2 (52.8–91.9)	50.1 (29.7–64.5)	0.024
Descending aorta	51.5 (37.2–107.0)	61.3 (40.4–107.4)	49.3 (15.6–99.6)	0.548
Mid descending aorta	58.8 (43.7–86.8)	60.4 (45.9–84.9)	56.5 (38.1–88.2)	0.725

Plaque severity was assessed by atheroma grade and CT-derived attenuation values. LAP was defined as 0–60 HU, and IAP as 61–130 HU. Data are presented as numbers (percentages) or medians (interquartile ranges).

HU: Hounsfield unit; NCIL: new cerebral infarction lesion; IQR: interquartile range; IAP: intermediate-attenuation plaque area; LAP: low-attenuation plaque

### Incidence and distribution of NCIL

**Fig. 2:**
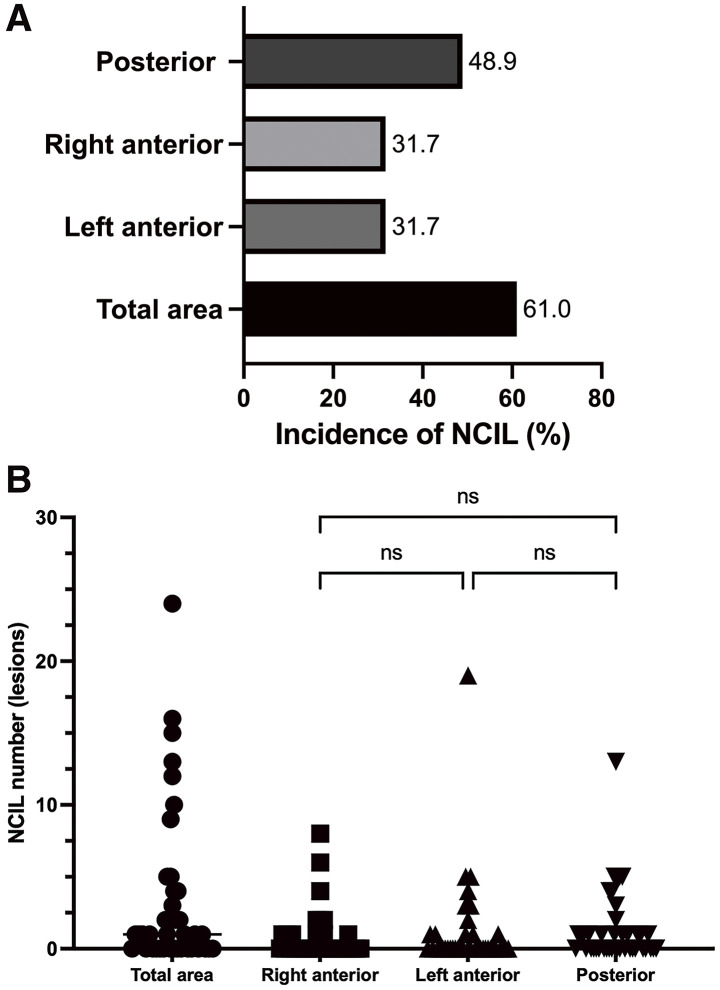
(**A**) Distribution of NCIL incidence across vascular territories. (**B**) Distribution of NCIL counts per vascular territory; no significant differences were observed. NCIL: new cerebral infarction lesion; ns: not significant

Postoperative DW-MRI identified NCILs in 25 patients (61.0%), of which 23 were silent and 2 were associated with symptomatic neurological dysfunction (**Table 3**). Lesions were most frequently located in the posterior circulation (48.9%), followed by the anterior circulation in both hemispheres (31.7% right, 31.7% left) (**Fig. 2A**). Fourteen patients (34.1%) had lesions in both the anterior and posterior territories, while 12.2% had anterior-only and 14.6% had posterior-only involvement. In total, 138 lesions were detected: 57 in the posterior, 49 in the left anterior, and 32 in the right anterior territory (**Fig. 2B**). No lesions demonstrated imaging features consistent with watershed or border-zone infarction, such as bilateral or cortical–subcortical lesions located at arterial boundary zones. The median interval to postoperative DW-MRI was 11 (IQR 8–13days).

**Table 3:** Incidence and distribution of NCILs

Variable	n = 41
Postoperative NCIL, n (%)	
Silent	23 (56.1)
Symptomatic neurological dysfunction	2 (4.9)
Distribution of NCIL in brain territory, n (%)	
Right anterior	13 (31.7)
Left anterior	13 (31.7)
Posterior	20 (48.9)
Bilateral anterior	7 (17.1)
Both anterior and posterior	14 (34.1)
Anterior only	5 (12.2)
Posterior only	6 (14.6)
NCIL number, lesions	
Total area	138
Right anterior area	32
Left anterior area	49
Posterior area	57

Data are presented as numbers (percentages) or lesion counts. Lesions were classified according to vascular territories (right anterior, left anterior, posterior circulation).

NCIL: new cerebral infarction lesion

### Operative details and early outcomes

Operative strategies, including the arterial inflow site, selective cerebral perfusion (95.1%), and deep hypothermic circulatory arrest (4.9%), were comparable between the groups (**Table 4**). Concomitant staged TEVAR was performed in 61.0% of patients, without differences between the NCIL-positive and negative groups.

**Table 4:** Operative details of patients with and without NCIL

Variable	All (n = 41)	NCIL-positive (n = 25)	NCIL-negative (n = 16)	*p* Value
Arterial perfusion, n (%)				
Right axillary artery and femoral artery	26 (63.4)	15 (60.0)	11 (68.8)	0.742
Right axillary artery	1 (2.4)	1 (4.0)	0 (0.0)	0.376
Ascending aorta and right axillary artery	5 (20.0)	4 (16.0)	1 (6.3)	0.376
Ascending aorta	4 (9.8)	3 (12.0)	1 (6.3)	1.00
Ascending aorta and femoral artery	3 (7.3)	1 (4.0)	2 (12.5)	0.550
Femoral artery	2 (4.9)	1 (4.0)	1 (6.3)	1.00
Cerebral perfusion, n (%)				
Selective cerebral perfusion	39 (95.1)	24 (96.0)	15 (93.8)	1.00
Deep hypothermic circulatory arrest	2 (4.9)	1 (4.0)	1 (6.3)	1.00
Second-stage TEVAR, n (%)	25 (61.0)	15 (60.0)	10 (62.5)	1.00
Operation time (min), median (IQR)	361 (316–456)	364 (331–456)	352 (306–466)	0.511
Cardiopulmonary bypass time (min), median (IQR)	200 (181–265)	203 (178–265)	194 (181–276)	1.00
Lower body circulatory arrest time (min), median (IQR)	49 (37–56)	50 (36–62)	46 (37–54)	0.432

Operative strategies and cardiopulmonary bypass variables are shown. Data are presented as numbers (percentages) or medians (interquartile ranges).

TEVAR: thoracic endovascular aortic repair; NCIL: new cerebral infarction lesion; IQR: interquartile range

No 30-day mortalities were observed (**Table 5**). Symptomatic stroke occurred in 2 patients (8.0%) in the NCIL-positive group, and paraparesis occurred in 3 patients (7.3%). The lengths of ICU and hospital stay were not significantly different between the groups.

**Table 5:** Early postoperative outcomes

Variable	All (n = 41)	NCIL-positive (n = 25)	NCIL-negative (n = 16)	*p* Value
30-day mortality, n (%)	0 (0.0)	0 (0.0)	0 (0.0)	N/A
Symptomatic stroke (modified Rankin Scale >2), n (%)	2 (4.9)	2 (8.0)	0 (0.0)	0.512
Intensive care unit stay (days), median (IQR)	4 (3–5)	5 (3–6)	3 (3–5)	0.082
Hospital stay (days), median (IQR)	21 (15–31)	18 (15–29)	19 (15–26)	0.841
Spinal cord ischemia, n (%)				
Paraplegia	0 (0.0)	0 (0.0)	0 (0.0)	N/A
Paraparesis	3 (7.3)	2 (8.0)	1 (6.3)	1.00
Heart failure, n (%)	0 (0.0)	0 (0.0)	0 (0.0)	N/A
Postoperative new-onset atrial fibrillation, n (%)	18 (43.9)	8 (32.0)	10 (62.5)	0.106
Tracheostomy, n (%)	0 (0.0)	0 (0.0)	0 (0.0)	N/A
Pancreatitis, n (%)	1 (2.4)	0 (0.0)	1 (6.3)	N/A
Worsening creatinine level >1, n (%)	1 (2.4)	1 (4.0)	0 (0.0)	N/A

Data are presented as numbers (percentages) or medians (interquartile ranges). Symptomatic stroke was defined as a postoperative neurological deficit with a modified Rankin Scale >2.

NCIL: new cerebral infarction lesion; IQR: interquartile range; N/A: not available

### Predictors of NCIL

Univariable logistic regression analysis identified atheroma grade in the aortic arch (OR, 2.23; 95% CI, 1.23–4.52; *p* = 0.014), atheroma grade in the mid-descending aorta (OR, 2.01; 95% CI, 1.19–3.71; *p* = 0.015), LAP area in the aortic arch (/10 mm^2^) (OR, 2.40; 95% CI, 1.48–5.44; *p* <0.01), and IAP area in the aortic arch (/10 mm^2^) (OR, 1.39; 95% CI, 1.07–1.94; *p* = 0.025) as significant predictors of NCIL (**Table 6**). In the multivariable model, the LAP area in the aortic arch (/10 mm^2^) remained an independent predictor of NCIL (OR, 3.01; 95% CI, 1.50–8.75; *p* = 0.012). Importantly, this finding was consistent across sensitivity analyses incorporating IAP and descending aortic variables, in which arch LAP retained its independent predictive value (**Supplementary Table 1**). In a sensitivity analysis restricted to patients managed with selective cerebral perfusion, excluding the 2 patients who underwent deep hypothermic circulatory arrest, the association between arch LAP area and NCIL remained significant in multivariable analysis (OR 2.77 per 10 mm^2^, 95% CI 1.36–8.21; *p* = 0.022) (**Supplementary Tables 2** and **3**).

**Table 6:** Univariable and multivariable logistic regression analyses for predictors of NCIL occurrence

	Univariable			Multivariable		
Variables	OR	95% CI	*p* Value	OR	95% CI	*p* Value
Age	1.02	0.94–1.10	0.64	0.96	0.86–1.06	0.47
Male	1.21	0.21–6.37	0.82	0.66	0.05–7.92	0.73
Degenerative	1.17	0.28–4.61	0.82			
Hypertension	2.65	0.39–22.19	0.32			
Hyperlipidemia	0.90	0.24–3.26	0.87			
Current smoker	0.59	0.13–2.31	0.46			
Previous stroke	2.86	0.37–58.90	0.33			
Coronary artery disease	2.85	0.37–58.90	0.33			
Peripheral artery disease	0.61	0.07–5.54	0.64			
Atheroma grade in aortic arch	2.23	1.23–4.52	0.014	1.09	0.30–3.96	0.89
Atheroma grade in mid DTA	2.01	1.19–3.71	0.015			
LAP area in the aortic arch (/10 mm^2^)	2.40	1.48–5.44	<0.01	3.01	1.50–8.75	0.012
Intermediate-attenuation plaque area in the aortic arch (/10 mm^2^)	1.39	1.07–1.94	0.025	0.83	0.50–1.31	0.429
Second-stage TEVAR	0.90	0.24–3.26	0.87			

OR and 95% CIs are shown. Variables with *p* <0.10 in univariable analysis were entered into the multivariable model.

NCIL: new cerebral infarction lesion; DTA: descending thoracic aorta; TEVAR: thoracic endovascular aortic repair; CI: confidence interval; OR: odds ratios

## Discussion

In the present study, postoperative DW-MRI revealed NCILs in 61% of patients undergoing TAR, the majority of which were clinically silent. This incidence was considerably higher than the reported frequency of symptomatic stroke (2%–16%), but aligns with prior MRI-based studies that systematically evaluated silent lesions.^1)^ Leshnower et al. documented a 70% incidence after hemiarch replacement,^17)^ and Hughes et al. demonstrated that postoperative MRI consistently detected brain injury regardless of temperature management, with associated structural, functional, and cognitive changes.^18)^ Together with DW-MRI studies in endovascular arch repair reporting NCIL rates of 26%–81%,^19–23)^ these findings highlight the underrecognized burden of silent cerebral injury in arch surgery when relying solely. However, it should be noted that the present cohort consisted exclusively of patients undergoing elective TAR with postoperative MRI evaluation and therefore may not fully reflect the incidence of NCILs in an unselected arch surgery population (**Supplementary Tables 4** and **5**).

Importantly, the LAP burden in the aortic arch was identified as the sole independent predictor of NCIL occurrence, whereas conventional atheroma grade failed to retain significance after adjustment. This finding is biologically plausible, given that LAP represents lipid-rich, rupture-prone plaque and is directly exposed to high-velocity and turbulent flow during TAR. The robustness of this association was confirmed by sensitivity analyses, in which arch LAP consistently remained an independent predictor of NCIL. Our findings are consistent with previous evidence linking LAP in the carotid and thoracic aorta with embolic complications,^15,16,24)^ and extend this concept to the setting of open arch replacement. Collectively, these results suggest that the embolic potential during TAR is shaped not only by intraoperative hemodynamic alterations but also by the intrinsic vulnerability of aortic arch plaques. However, the estimated effect size was associated with considerable uncertainty, as reflected by the relatively wide 95% confidence interval (OR 3.01 per 10 mm^2^; 95% CI 1.50–8.75), likely due to the limited number of events and potential model instability; therefore, the magnitude of the association should be interpreted cautiously.

Although the imaging features of NCILs were consistent with an embolic distribution, the embolic source cannot be attributed exclusively to aortic arch plaque. Other potential sources of cerebral embolism during TAR include carotid or vertebral artery atherosclerosis, intraoperative air embolism, and embolization related to cannulation or manipulation of the ascending aorta. Therefore, the observed association between arch LAP burden and NCIL should be interpreted within a multifactorial framework, in which vulnerable arch plaque represents a major, but not exclusive, contributor to perioperative cerebral embolism. Accordingly, our findings demonstrate an association rather than proof of a direct causal relationship.

From a clinical standpoint, CT-derived quantification of LAP in the aortic arch may represent a promising instrument for preoperative risk stratification. In contrast to morphology-based grading systems that primarily capture plaque thickness or mobility, LAP assessment provides insight into plaque composition and may more accurately reflect embolic vulnerability. In the present cohort, the median arch LAP burden was approximately 25 mm^2^, whereas an exploratory receiver-operating characteristic analysis suggested a higher threshold, on the order of 40 mm^2^, for discriminating patients at increased risk of NCIL. These values should, however, be interpreted as hypothesis-generating rather than prescriptive, given the single-center nature of the study and the limited sample size.

Integration of LAP quantification into operative planning could therefore facilitate risk-adapted strategies aimed at mitigating embolic load, including minimization of aortic manipulation, thoughtful selection of arterial inflow configuration, or intensification of cerebral protective measures. Pending external validation, such thresholds might also assist in identifying patients who could be considered for advanced neuroprotective approaches, including brain isolation techniques, within a personalized perioperative management paradigm.

Future multicenter investigations will be essential to establish the reproducibility, calibration, and clinical utility of LAP-guided decision frameworks and to incorporate plaque composition into comprehensive models of perioperative cerebral risk.

This study has several limitations. First, the sample size was modest, limiting statistical power and generalizability. Second, patients unable to undergo MRI (e.g., emergent cases or those requiring concomitant functional brain isolation) were excluded, potentially leading to selection bias. Because these patients typically represent a higher-risk population for cerebral embolic events, the present results may underestimate the true incidence of NCILs and are primarily applicable to elective TAR cases. In addition, staged TEVAR may represent a potential confounder when evaluating cerebral infarction after TAR, given the embolic risk associated with endovascular manipulation. To address this issue, we performed a sensitivity analysis restricted to a pure TAR cohort managed with selective cerebral perfusion. Although this subgroup analysis was underpowered, the direction and magnitude of the association between arch LAP burden and NCIL were generally consistent with those observed in the primary model, suggesting that the main findings are unlikely to be driven solely by subsequent endovascular intervention.

Third, the retrospective, single-center design may limit external validity. Fourth, postoperative MRI was performed within a relatively broad window (5–14 days; median 11 days). This timing may have led to (i) underdetection of very early transient diffusion-restricted lesions that can partially resolve over time, and (ii) failure to capture late-onset infarctions occurring after the MRI.

Fifth, neurocognitive outcomes were not systematically assessed, and thus the functional consequences of silent lesions could not be determined. Sixth, plaque analysis relied on CT attenuation thresholds, which, although standardized, cannot fully capture plaque biology without histopathological correlation. Finally, arterial inflow strategies were selected according to an institutional algorithm; however, they were not completely uniform across patients. Differences in cannulation configuration may have resulted in variability in cerebral perfusion conditions despite a common systemic temperature target and therefore represent a potential source of residual confounding.

These limitations should be considered when interpreting the findings.

## Conclusion

DW-MRI demonstrated that more than half of patients undergoing TAR developed NCILs, predominantly silent. A greater preoperative CT-derived LAP burden in the aortic arch was independently associated with the occurrence of NCIL, highlighting the potential importance of plaque composition beyond conventional morphology-based assessment. These findings underscore the underrecognized burden of silent cerebral injury after arch surgery and suggest that arch LAP assessment may contribute to perioperative risk stratification and the development of tailored neuroprotective strategies within a multifactorial embolic framework.

## Declarations

### Data availability

The data supporting the findings of this study are available from the corresponding author upon reasonable request.

## Supplementary Information

Supplemental Table 1: Sensitivity analyses for predictors of NCIL occurrence. Different model specifications consistently demonstrated the independent predictive value of aortic arch LAP.

Supplementary Table 2: Logistic regression analysis restricted to patients managed with selective cerebral perfusion. To account for heterogeneity in cerebral protection strategy, analyses were repeated after excluding the 2 patients who underwent deep hypothermic circulatory arrest.

Supplementary Table 3: Logistic regression analysis in patients undergoing selective cerebral perfusion without staged TEVAR (pure TAR cohort). This subgroup analysis was performed to evaluate whether the association between arch LAP burden and NCIL could be explained by subsequent endovascular intervention.

Supplementary Table 4: Baseline characteristics of the MRI cohort compared with patients excluded from MRI analysis. Clinical and anatomical variables are summarized for patients included in the MRI cohort and for major excluded groups, including those treated with functional brain isolation and those excluded for predefined reasons (e.g., MRI contraindications).

Supplementary Table 5: Perioperative characteristics and outcomes of patients excluded from MRI analysis. Objective perioperative variables and early outcomes are presented for excluded patients, including emergency/urgent cases and those managed with functional brain isolation.
