# Supplementary material for: CT-Derived Aortic Plaque Characteristics Predict MRI-Detected Silent Cerebral Infarction after Total Aortic Arch Replacement
Source: Ann Thorac Cardiovasc Surg. 2026 Apr 14;32(1):25-00215. doi: 10.5761/atcs.oa.25-00215 (PMC13082872; doi:10.5761/atcs.oa.25-00215)
Supplement: Supplemental Table 1: — Sensitivity analyses for predictors of NCIL occurrence. Different model specifications consistently demonstrated the independent predictive value of aortic arch LAP. [file atcs-32-1-25-00215-s001.pdf]

Supplemental Table 1

Model specification	Variables included	OR for LAP aortic arch (95% CI)	*p* value
Main model	Arch atheroma grade, LAP aortic arch, IAP aortic arch, Age, Sex	2.60 (1.44–6.81)	0.012
+ Mid DTA atheroma grade	Arch atheroma grade, LAP aortic arch, Mid DTA grade, Age, Sex	2.67 (1.44–7.44)	0.015
LAP as tertiles	Arch LAP (tertiles), Arch atheroma grade, Age, Sex	High vs low: 13.12 (2.14–124.58)	< 0.01
Excluding symptomatic strokes (n=39)	Arch atheroma grade, Arch LAP, Age, Sex	2.59 (1.43–6.80)	0.013
+ Adjusted for staged TEVAR	Arch atheroma grade, LAP aortic arch, Age, Sex, TEVAR	2.61 (1.43–6.92)	0.013

CI, confidence interval; DTA, descending thoracic aorta; IAP, intermediate attenuation plaque; LAP, low attenuation plaque; OR, odds ratio; TEVAR, thoracic endovascular aortic repair

Supplemental Table 2

Logistic regression analysis restricted to patients managed with selective cerebral perfusion

	Univariable			Multivariable		
Variables	OR	95% CI	*p* values	OR	95% CI	*p* values
Age	1.02	0.94–1.10	0.659	0.97	0.86–1.07	0.524
Male	1.25	0.22–6.65	0.792	0.68	0.05–8.19	0.754
Degenerative	1.21	0.29–4.88	0.784			
Hypertension	2.75	0.40–23.17	0.302			
Hyperlipidemia	1.11	0.29–4.19	0.876			
Current smoker	0.61	0.14–2.40	0.487			
Previous stroke	2.80	0.36–57.93	0.379			
Coronary artery disease	2.80	0.36–57.93	0.379			
Peripheral artery disease	0.59	0.06–5.41	0.619			
Atheroma grade aortic arch	2.39	1.27–5.18	0.013	1.13	0.32–4.09	0.848
Atheroma grade in mid DTA	1.93	1.11–3.67	0.028			
Low attenuation plaque area aortic arch (/10 mm^2)	2.54	1.49–6.42	< 0.01	2.77	1.36–8.21	0.022
Intermediate attenuation plaque area aortic arch (/10 mm^2)	1.55	1.14–2.34	0.015	0.92	0.54–1.54	0.746
Second staged TEVAR	0.93	0.24–3.47	0.918			

CI, confidence interval; DTA, descending thoracic aorta; OR, odds ratio; TEVAR, thoracic endovascular aortic repair

Supplementary Table 3.

Logistic regression analysis in patients undergoing selective cerebral perfusion without staged TEVAR (pure TAR cohort)

Variables	OR	95% CI	*p* values	OR	95% CI	*p* values
Age	1.04	0.95–1.16	0.398	0.99	0.89–1.11	0.883
Male	0.80	0.03–10.72	0.869			
Degenerative	3.00	0.38–30.47	0.309			
Hypertension	NA	NA	NA			
Hyperlipidemia	4.00	0.45–45.17	0.223			
Current smoker	0.33	0.03–2.60	0.309			
Previous stroke	NA	NA	NA			
Coronary artery disease	NA	NA	NA			
Peripheral artery disease	NA	NA	NA			
Atheroma grade aortic arch	2.53	0.93–11.15	0.124			
Atheroma grade in mid DTA	1.83	0.80–5.72	0.203			
Low attenuation plaque area aortic arch (/10 mm^2)	1.82	1.10–4.40	0.070	1.85	1.07–4.71	0.081
Intermediate attenuation plaque area aortic arch (/10 mm^2)	1.73	0.96–4.49	0.137			

Because of the small sample size and sparse outcome events, several binary variables exhibited complete or quasi-complete separation. Therefore, reliable effect estimates could not be obtained and are shown as not estimable.

CI, confidence interval; DTA, descending thoracic aorta; OR, odds ratio; TEVAR, thoracic endovascular aortic repair

Supplemental Table 4

Baseline characteristics of the MRI cohort compared with patients excluded from MRI analysis

Variable	All (n=41)	Brain isolation (n=8)	Excluded cohort (n=6)
Age, yrs median (IQR)	77 (73–79)	78.5 (72.0–82.0)	75.0 (73.0–76.0)
Male, n (%)	34 (82.9)	8.0 (100.0%)	6.0 (100.0%)
BSA, m^2 median (IQR)	1.66 (1.53–1.75)	1.7 (1.6–1.8)	1.8 (1.7–1.8)
Hypertension	36 (87.8)	8.0 (100.0%)	5.0 (83.3%)
Hyperlipidemia	25 (61.0)	7.0 (87.5%)	4.0 (66.7%)
Diabetes mellitus	8 (19.5)	1.0 (12.5%)	2.0 (33.3%)
Current smoker	28 (68.3)	6.0 (75.0%)	6.0 (100.0%)
COPD	16 (39.0)	5.0 (62.5%)	3.0 (50.0%)
Past stroke	5 (12.2)	2.0 (25.0%)	1.0 (16.7%)
Coronary artery disease	5 (12.2)	1.0 (12.5%)	1.0 (16.7%)
Peripheral artery disease	4 (9.8)	1.0 (12.5%)	0.0 (0.0%)
Cancer	3 (7.3)	2.0 (25.0%)	1.0 (16.7%)
LVEF, % median (IQR)	64.0 (60.0–70.8)	62.0 (55.5–71.0)	65.5 (59.0–69.0)
Hemoglobin, g/dL median (IQR)	12.9 (12.2–14.0)	14.3 (13.1–15.5)	14.1 (12.9–15.4)
eGFR, mL/min/1.73m² median (IQR)	57.2 (47.3–73.6)	42.9 (35.5–55.9)	43.3 (32.7–57.9)
Albumin, g/dL median (IQR)	4.1 (3.9–4.2)	4.1 (3.7–4.3)	3.9 (3.2–4.3)
Anti-platelet	8 (19.5)	2.0 (25.0%)	4.0 (66.7%)
Statin	19 (46.3)	6.0 (75.0%)	4.0 (66.7%)
Degenerative aneurysm	29 (70.7)	8.0 (100.0%)	5.0 (83.3%)
Dissection	12 (29.3)	0.0 (0.0%)	0.0 (0.0%)
Aneurysm diameter, mm median (IQR)	55.0 (53.0–62.5)	57.5 (52.8–76.8)	58.0 (56.0–60.0)
Bovine arch	1 (2.4)	2.0 (28.6%)	1.0 (16.7%)

Excluded cohort indicates patients excluded from MRI analysis for non-emergent reasons (e.g., MRI contraindications such as implanted devices, non-neurological early death, or refusal).

Supplemental Table 5

Baseline and perioperative characteristics of patients excluded from the MRI analysis

Variable	All (n=41)	Emergent or urgent cases (n=27)	Brain isolation (n=8)	Excluded cohort (n=6)
Age, yrs median (IQR)	77 (73–79)	68.0 (63.0–75.0)	78.5 (72.0–82.0)	75.0 (73.0–76.0)
Male, n (%)	34 (82.9)	16 (59.3)	8 (100.0%)	6.0 (100.0%)
Hypertension, n (%)	36 (87.8)	23 (85.2)	8 (100.0%)	5.0 (83.3%)
Hyperlipidemia, n (%)	25 (61.0)	10 (37.0)	7 (87.5%)	4.0 (66.7%)
Diabetes mellitus, n (%)	8 (19.5)	4 (14.8)	1 (12.5%)	2.0 (33.3%)
Preoperative impaired consciousness, n (%)	0 (0.0)	1 (3.7)	0 (0.0)	0 (0.0)
Operation time, min, median (IQR)	361 (316-456)	476 (356–543)	386 (332–465)	393 (350–464)
30-day mortality, n (%)	0 (0.0)	1 (3.7)	0 (0.0)	1 (16.7)
Symptomatic stroke (Modified Rankin scale >2), n (%)	2 (4.9)	5 (18.5)	0 (0.0)	0 (0.0)
Hospital stay, day, median (IQR)	21 (15-31)	38 (24–61)	32 (20–55)	15 (11–85)
Spinal cord ischemia, n (%)				
Paraplegia	0 (0.0)	0 (0.0)	1 (12.5)	0 (0.0)
Paraparesis	3 (7.3)	0 (0.0)	0 (0.0)	0 (0.0)
Tracheostomy, n (%)	0 (0.0)	5 (18.5)	2 (25.0)	0 (0.0)

Excluded cohort indicates patients excluded from MRI analysis for non-emergent reasons (e.g., MRI contraindications such as implanted devices, non-neurological early death, or refusal).

Emergent/urgent includes rupture, acute dissection, and mycotic aneurysm.
